# Periodontitis and Glycaemic Dyscontrol Substantially Increase the Risk of Medication-Related Osteonecrosis of the Jaw: A Preclinical Study in Female Rats

**DOI:** 10.3390/jcm15176799

**Published:** 2026-09-02

**Authors:** João Martins de Mello-Neto, Luan Felipe Toro, Letícia Chaves Ferreira, Vinícius Franzão Ganzaroli, Leandro Lemes da Costa, Rodrigo Isaías Lopes Pereira, Arthur Michellim Kirasuke, Ana Beatriz Carreto, Isabella Zacarin Guiati, Valdir Gouveia Garcia, Letícia Helena Theodoro, Edilson Ervolino

**Affiliations:** 1 College of Medicine and Dentistry, James Cook University, Cairns, QLD 4870, Australia; joao.martinsdemelloneto@jcu.edu.au; 2 Department of Basic Sciences, School of Dentistry, São Paulo State University (UNESP), Araçatuba 16015-050, SP, Brazil; luan.toro@unesp.br (L.F.T.); leticia@cairnsspecialistdental.com.au (L.C.F.); vinicius.ganzaroli@unesp.br (V.F.G.); leandro.lemes@unesp.br (L.L.d.C.); rodrigo.l.pereira@unesp.br (R.I.L.P.); arthur.m.kirasuke@unesp.br (A.M.K.); ab.carreto@unesp.br (A.B.C.); izguiati@usp.br (I.Z.G.); 3 Department of Basic Subjects, Marília Medical School (FAMEMA), Marília 17519-030, SP, Brazil; 4 Department of Biomaterials and Oral Biology, School of Dentistry, University of São Paulo, São Paulo 05508-000, SP, Brazil; 5 Latin American Institute of Dental Research and Education (ILAPEO), Curitiba 80810-030, PR, Brazil; valdir.garcia@unesp.br; 6 Department of Diagnostic and Surgery, School of Dentistry, São Paulo State University (UNESP), Araçatuba 16015-050, SP, Brazil; leticia.theodoro@unesp.br

**Keywords:** bisphosphonates, diabetes mellitus, MRONJ, periodontitis, zoledronate

## Abstract

**Background/Objectives**: There is limited understanding of how periodontitis progresses in individuals with diabetes who are undergoing antiresorptive drug therapy, particularly given that both are risk factors for medication-related osteonecrosis of the jaw (MRONJ). This study evaluated the effect of therapy with an oncological dose of zoledronate on the progression of experimental periodontitis (EP) in diabetic female rats. **Methods**: One hundred and eight female rats (12 months old) were assigned to four groups: VEH-NG (n = 26), VEH-DM (n = 28), ZOL-NG (n = 26) and ZOL-DM (n = 28). VEH-NG and VEH-DM groups received 0.45 mL of vehicle, and ZOL-NG and ZOL-DM groups received 0.45 mL of zoledronate (100 μg/kg). Treatment with vehicle or zoledronate was administered every three days for seven weeks. At week 0, a cotton ligature was placed around the right and left mandibular first molars to induce experimental periodontitis (EP). In week 2, VEH-DM and ZOL-DM groups were subjected to an injection of streptozotocin into the caudal vein to induce diabetes mellitus (DM). VEH-NG and ZOL-NG groups received sodium citrate buffer via the same route. Euthanasia was performed 14, 21, and 35 days after ligature placement. Microtomographic, histomorphometric, and immunohistochemical analyses (TNFα, IL-1β, and TRAP) were performed. **Results**: The percentage of non-vital bone tissue, local inflammation, and TNFα and IL-1β immunolabelling in the furcation region were higher in ZOL-NG and even higher in ZOL-DM. Alveolar bone loss and the number of TRAP-positive cells were lower in the groups treated with zoledronate. **Conclusions**: During treatment with an oncological dose of zoledronate, periodontitis and glycaemic dyscontrol substantially increase the risk of MRONJ.

## 1. Introduction

Bisphosphonates and receptor activator of nuclear factor-kappa B ligand (RANKL) inhibitors, such as denosumab, are antiresorptive drugs used in the treatment of bone loss or lesions due to osteopenia/osteoporosis, multiple myeloma, Paget’s disease, osteogenesis imperfecta, and to prevent the progression of bone metastases, reduce bone pain and hypercalcaemia of malignancy [1]. However, one of the adverse effects of this class of drugs is medication-related osteonecrosis of the jaw (MRONJ) [1].

MRONJ is a debilitating condition defined as exposed bone or a bone that can be probed through an intraoral or orocutaneous fistula and has persisted for more than eight weeks in patients with no history of radiation therapy to the jaws or metastatic disease of the jaw with a history of receiving antiresorptive and/or antiangiogenic agents [1]. The etiopathogenesis of MRONJ has not yet been fully elucidated [2]. However, the risk factors related to the disease are well established [1,2,3]. It has been observed that the prevalence of MRONJ is higher in elderly women [3,4,5]. The use of more potent antiresorptive drugs and high doses to complement cancer therapy is related to most cases. Invasive dental procedures, such as tooth extraction, and infectious/inflammatory diseases related to the tooth, such as periodontitis, are among the main local risk factors. In addition, the presence of comorbidities, such as diabetes mellitus (DM), is also an important aggravating factor [3,4,5].

The prevalence of both periodontitis and DM has progressively increased worldwide [6,7,8]. Periodontitis and DM have a well-established bidirectional relationship [9,10]. Periodontitis increases inflammatory cytokine levels, which may be exacerbated in the presence of DM and contribute to insulin receptor desensitisation, thereby impairing glycaemic control [11]. Conversely, chronic hyperglycaemia has been associated with the production of Advanced Glycation End Products (AGEs) and inflammatory cytokines, which can increase the severity of periodontitis [11,12]. As life expectancy increases worldwide, age-related diseases requiring antiresorptive drug therapy are becoming more common. Therefore, it is important to understand the effects of periodontitis in patients with DM who are undergoing antiresorptive drug therapy, particularly for MRONJ prevention [13]. We hypothesise that, during treatment with an oncological dose of zoledronate, the combination of periodontitis and uncontrolled glycaemia substantially increases the risk of MRONJ. Thus, this study aimed to analyse the progression of experimental periodontitis (EP) during treatment with an oncological dose of zoledronate in diabetic female rats.

## 2. Materials and Methods

### 2.1. Animals, Ethical Considerations, Sample Size Calculation, and Randomisation

This research followed ARRIVE (Animal Research: Reporting of in vivo Experiments) [14]. The present study used one hundred and eight 12-month-old female rats (Wistar—Rattus norvegicus) supplied by the Animal Resources Centre of the School of Dentistry, São Paulo State University (FOA-UNESP) with body weights ranging from 350 to 500 g. Housing conditions included a 12-h light/dark cycle, an ambient temperature of 22 ± 2 °C, a ventilation and exhaust system providing 20 air exchanges per hour, and a relative humidity of approximately 55 ± 5%. The animals were kept in plastic cages (4 animals per cage) with ad libitum access to food and water. All appropriate measures were taken to minimise the number of animals used and to prevent suffering. Experimental handling procedures were performed in accordance with the standards established by the National Council for the Control of Animal Experimentation (CONCEA), and the experimental protocol was approved by the Ethics Committee on Animal Use (CEUA) of FOA-UNESP (#005332016).

The sample size was based on the study by Nunes et al. [15] that employed ligature-induced periodontitis in diabetic rats. The primary endpoint used for this calculation was the alveolar bone present in the periodontium related to the first molar, assessed by histometric analysis, with four experimental groups, a significance level of 5%, a statistical power of 95%, and two-way ANOVA analysis. Based on the means and measures of dispersion observed in the study by Nunes et al. [15] (overall mean ≈ 0.100 mm^2^; pooled standard deviation ≈ 0.030 mm^2^), an effect size of f ≈ 0.8 was estimated, resulting in 8 animals per experimental period; that is, 24 animals per group. Accounting for potential animal losses during the experiment, the VEH-NG and ZOL-NG groups consisted of 26 animals each, while the VEH-DM and ZOL-DM groups consisted of 28 animals each, given the higher rate of loss associated with DM. Thus, the total number of animals at the start of the experiment was 108.

This study followed a controlled, randomised, and blinded design. Each animal was identified by a numerical sequence from 1 to 108. Minitab^®^ 17 software (Minitab Inc., State College, PA, USA) was used to generate a table containing the randomised distribution of the numbered animals into the different experimental groups.

### 2.2. Study Design

During procedures that could involve pain or discomfort, such as ligature placement, tail-vein injection of solutions, and euthanasia, the animals were anaesthetised with an intramuscular injection of ketamine hydrochloride (80 mg/kg) (Cetamim, Syntec, Santana do Parnaíba, SP, Brazil) and xylazine hydrochloride (10 mg/kg) (Xilazin, Syntec, Santana do Parnaíba, SP, Brazil).

**Figure 1:**
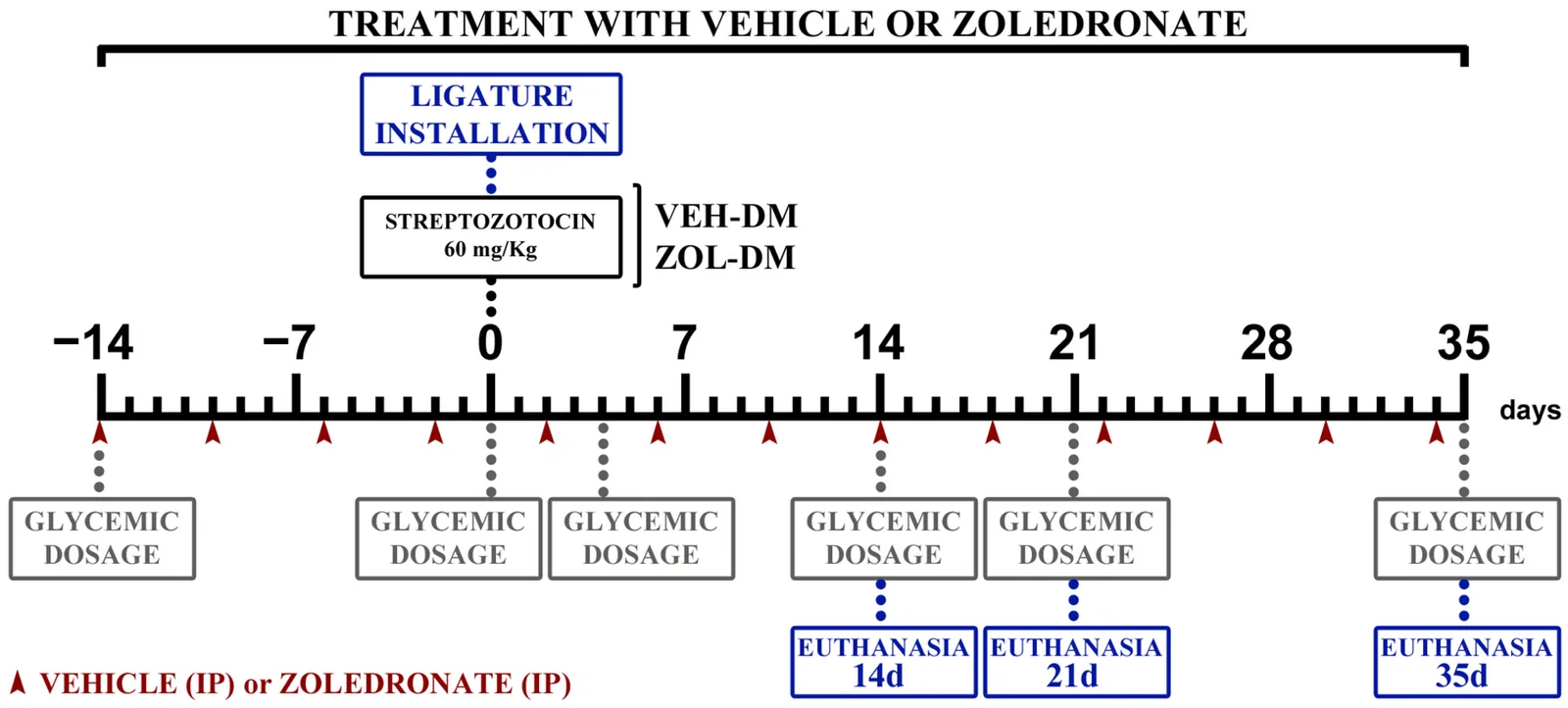
Schematic illustration depicting the experimental design and the experimental procedures performed at their respective time intervals in the VEH-NG, VEH-DM, ZOL-NG, and ZOL-DM groups.

Figure 1 depicts the study design. One hundred and eight female rats (12 months old) were used for the experiment. The selection of 12-month-old female rats was based on epidemiological studies on MRONJ, which indicate a higher prevalence of the disease among middle-aged and elderly women [4,5]. Considering the physiological and metabolic characteristics of the species, as well as the duration of the experimental protocol, this animal model approximately corresponds to middle-aged to senescent women [16].

The female rats were randomly assigned to the following groups: VEH-NG (n = 26; treated with vehicle and normoglycaemic), VEH-DM (n = 28; treated with vehicle and with diabetes mellitus), ZOL-NG (n = 26; treated with zoledronate and normoglycaemic), and ZOL-DM (n = 28; treated with zoledronate and with diabetes mellitus).

VEH-NG and VEH-DM groups received 0.45 mL of vehicle (physiological saline solution), while ZOL-NG and ZOL-DM groups received 0.45 mL of vehicle with an additional 100 µg/kg of zoledronate (Sigma, Saint Louis, MO, USA) [17,18,19]. Injections were given intraperitoneally every 3 days for 7 weeks (0–7th week). The dosage and treatment plan for the rats were adapted from the protocol currently used for humans to complement cancer therapy.

In the second week, 14 days after initiation of the drug treatment protocol, a cotton thread (Chain Cotton No. 24, Coats Corrente, São Paulo, SP, Brazil) was placed supragingivally, in close contact with the gingival margin, around the right and left mandibular first molars to induce biofilm accumulation and experimental periodontitis (EP) [20,21]. Throughout the experimental period, the ligature retention was monitored every other day.

Also in the second week, VEH-NG and ZOL-NG groups received 0.3 mL of sodium citrate buffer solution (Sigma, Saint Louis, MO, USA) (0.1 M; pH 4.0), while VEH-DM and ZOL-DM groups received 0.3 mL of the same solution with an additional 60 mg/kg of streptozotocin (Sigma, Saint Louis, MO, USA), both via the tail vein, to induce DM [22,23]. The animals’ glycaemic level was periodically monitored throughout the experimental period using the Accu-Check Active glucometer (Roche Diabetes Care, São Paulo, Brazil). Those animals with a fasting (12 h) glycaemic index greater than 200 mg/dL were considered diabetic and included in the study.

In each experimental group, there were three euthanasia periods: 14th, 21st and 35th days after the ligature was placed, which correspond to the fourth, fifth and seventh week of the experiment, respectively. These time points were selected to evaluate periodontal tissue alterations during early (14 days), intermediate (21 days), and later (35 days) stages of experimental periodontitis progression [20,24]. Animals were euthanised using transcardiac perfusion with 0.9% sodium chloride solution plus 0.1% heparin (100 mL), followed by a fixative solution (800 mL) consisting of 4% formaldehyde in phosphate-buffered saline, 0.1 M, 4 °C, pH 7.4 (Sigma, Saint Louis, MO, USA). The hemimandibles were carefully dissected and subjected to post-fixation in the same fixative solution for 72 h.

### 2.3. Sample Processing

The right hemimandibles of animals euthanised in the seventh week (35th day after the ligature placement) were submitted to micro-CT analysis. The left hemimandibles of all groups and experimental periods were demineralised in PBS plus 10% ethylenediaminetetraacetic acid (EDTA) for 90 days (Sigma, Saint Louis, MO, USA). The samples were then dehydrated, diaphanized, impregnated, embedded in paraffin and sectioned using a microtome (Leica Biosystems, Nussloch, Baden-Württemberg, Germany) at 4 μm thickness. The microtomy followed a cutting plane proceeding from buccal to lingual. Serial sections were collected from the furcation region of the lower left first molar. The histological sections were divided into two batches. One batch was subjected to haematoxylin-eosin staining for histopathological and histometric analysis, and the other for immunohistochemical analysis.

The histological sections allocated for immunohistochemical analyses were subdivided into three batches and subjected to the indirect immunoperoxidase technique following the protocol described by Souza et al., 2024 [25]. In summary: antigen retrieval was performed in citrate buffer in a pressurised chamber at 95 °C for 10 min (Decloaking chamber, Biocare Medical, Pacheco, CA, USA); endogenous peroxidase was blocked using 1.5% hydrogen peroxide (Sigma, Saint Louis, MO, USA) for 1 h; nonspecific sites were blocked using 3% bovine serum albumin (Sigma, Saint Louis, MO, USA) for 24 h; primary antibody incubation, rabbit anti-tumour necrosis factor-alpha (TNFα) antibody (orb11495, 1:100; Biorbyt, Cambridge, UK); rabbit interleukin 1-beta (IL1-β) antibody (orb382131, 1:100; Biorbyt, Cambridge, UK); mouse tartrate-resistant acid phosphatase (TRAP) antibody (sc376875, 1:200; Santa Cruz Biotechnology, Dallas, TX, USA) was performed for 24 h; biotinylated horse anti mouse/rabbit IgG antibody (1:100; Vector Laboratories, Newark, CA, USA) incubation was performed for 2 h; treatment with HRP-conjugated streptavidin (1:100; Vector Laboratories, Newark, CA, USA) was performed for 2 h; treatment with DAB (ImmPACT DAB Substrate kit, peroxidase, Vector Laboratories, Newark, CA, USA) was performed for 60 s for TNFα and IL-1β and for 30 s for TRAP; counterstaining was performed with Harris haematoxylin (Sigma, Saint Louis, MO, USA) for 2 min. As a negative control, the specimens were subjected to the previously described procedures without using the primary antibody.

### 2.4. Microtomography Analyses

The microtomography analyses were performed by an examiner blinded (JMMN) to the experimental group allocation. Specimens were identified by coded alphanumeric labels, and the examiner had no access to information regarding group/experimental period during image acquisition and analysis.

The right hemimandibles of animals euthanised on the 35th day after the ligature placement were analysed using the Micro-CT system (SkyScan 1272, Bruker, Billerica, MA, USA). The samples were scanned with an isotropic voxel size of 9 × 9 × 9 μm, 50 kVp, a rotation angle of 0.5°, a beam current of 500 μA, an aluminium filter of 0.5 mm, and an exposure time of 540 milliseconds. The NRECON^®^ software (software program version 1.6.3.3) was used to reconstruct the images of each sample. Linear measurements (coronal, sagittal and transaxial axes) in all three spatial dimensions were visualised and evaluated using the DataViewer^®^ program (software version 1.5.0). Finally, all images were oriented and saved in coronal slices (2000 × 1336). To determine the central region of the furcation, a 3D ROI (region of interest) was selected using the CTAn^®^ software program version 1.10.11.0. The examiner used morphological landmarks to guide the ROI design until the mesial and distal pulp of the lower first molar was identified. After identification, 30 sections (15 anterior and 15 posterior) were selected from the central region of the furcation for microtomographic analysis. Volumetric measurements were then performed. The contours were drawn below the cementoenamel junction (CEJ). They extended 1 mm towards the apical direction to ensure that the entire central area of the furcation region of the first molar was included in the Region of Interest (ROI). The following architectural parameters were evaluated: bone volume/total volume in the furcation region (BV/TV—%), thickness of trabeculae in the bone tissue of the furcation region (Tb.Th—mm), and number of trabeculae in the bone tissue of the furcation region (Tb.N—/mm). To analyse the level of alveolar bone loss using the DataViewer^®^ program, two linear measurements were taken from the cementoenamel junction to the alveolar bone crest (CEJ-ABC) in the mesial and distal directions.

### 2.5. Histopathological Analysis

The histopathological analysis was performed by an examiner blinded (E.E.) to the experimental group allocation. Slides were identified by coded alphanumeric labels, and the examiner had no access to information regarding group/experimental period during analysis. The following parameters were evaluated: intensity of the local inflammatory response, extension of the inflammatory process, alveolar bone resorption, and structuring pattern of connective tissue in the furcation region [26,27] (Table 1).

### 2.6. Histometric Analysis

The histometric analysis was performed by an examiner blinded (JMMN) to the experimental group allocation. Slides were identified by coded alphanumeric labels, and the examiner had no access to information regarding group/experimental period during analysis.

The Percentage of Bone Tissue in the furcation region (PBTF) was measured using an image analysis system (Axiovision 4.8.2, Carl Zeiss MicroImaging GmbH, 07740, Jena, Germany). The total furcation area (TFA) was measured, and then the bone tissue in the furcation area (BTFA) was measured, both in mm2. The PBTF was subsequently calculated by multiplying BTFA by 100 and dividing by TFA (PBTF = (BTFA × 100)/TFA). PBTF was expressed as % and as mean ± standard deviation in each experimental group.

The analysis system described above was used to determine the Percentage of Non-Vital Bone Tissue in the furcation region (PNVBT) of the first molars. The non-vital bone tissue areas (NVBTA) were measured in the furcation area, in mm2. PNVBT was calculated by multiplying NVBTA by 100 and dividing by BTFA (PNVBT = (NVBTA × 100)/BTFA). PNVBT was expressed as % and as mean ± standard deviation in each experimental group.

### 2.7. Immunolabelling Analysis of TNFα, IL-1β and TRAP

The immunohistochemical analysis was performed by examiners blinded to the experimental group allocation. Histological slides were identified by coded alphanumeric labels, and the examiners had no access to information regarding group/experimental period during analysis.

Images were obtained using a digital camera (AxioCam MRc5, Carl Zeiss, Göttingen, Germany) coupled to an optical microscope (AxioLab A1, Carl Zeiss, Göttingen, Germany) and connected to a microcomputer. Images measuring 1440 μm × 1080 μm were obtained in the furcation region of the first lower molar with EP for the immunohistochemical analysis of TNFα and IL-1β. These images were obtained in the centre of the furcation region. Its coronal limit was defined as the base of the furcation, and an apical limit extending 1440 μm. To assess the density of immunolabelling, the Colour Threshold tool in ImageJ software (version 1.x) was used to demarcate immunolabelling. The results were expressed as a percentage (%) as the mean ± standard deviation for each group. For the immunohistochemical analysis of TRAP, images measuring 720 μm × 540 μm were obtained in the furcation region of the first lower molar with EP. These images were centred in the furcation region, with the coronal limit set at the alveolar bone crest. The measurement extended 720 μm apically from this point. TRAP-positive multinucleated cells, as well as those attached to the bone matrix, were quantified. The results were expressed as the number of cells per square millimetre (/mm^2^) and presented as mean ± standard deviation for each group.

### 2.8. Statistical Analysis

Data were analysed using the Bioestat 5.3 program (Instituto Mamiruá, Manaus, AM, Brazil). The primary outcome in the present study was PNVBT. The remaining variables were considered as secondary outcomes. For CEJ-ABC, BV/TV, Tb.Th, Tb.N, PBTF, PNVBT, TNFα, IL-1β and TRAP, Levene’s test and the Shapiro–Wilk test were used. Two-way ANOVA, followed by the Tukey post-test, was performed. For histopathological analyses, the Kruskal–Wallis analysis and Student–Newman–Keuls post-test were used. The significance level adopted was 5% (*p* < 0.05).

## 3. Results

### 3.1. Animals and Clinical Findings

Ninety-six female rats were included in the study; that is, twenty-four animals in each group and eight animals in each experimental period. Twelve female rats were excluded because they did not meet the inclusion criteria. Ligature loss resulted in the exclusion of one animal from the VEH-NG group, one from the ZOL-NG group, and one from the ZOL-DM group. Among the animals undergoing DM induction, one animal from the VEH-DM group and three from the ZOL-DM group failed to reach the minimum blood glucose level required to continue in the experiment. Death before the end of the experiment occurred in one animal from the VEH-NG group, three from the VEH-DM group, and one from the ZOL-NG group.

**Figure 2:**
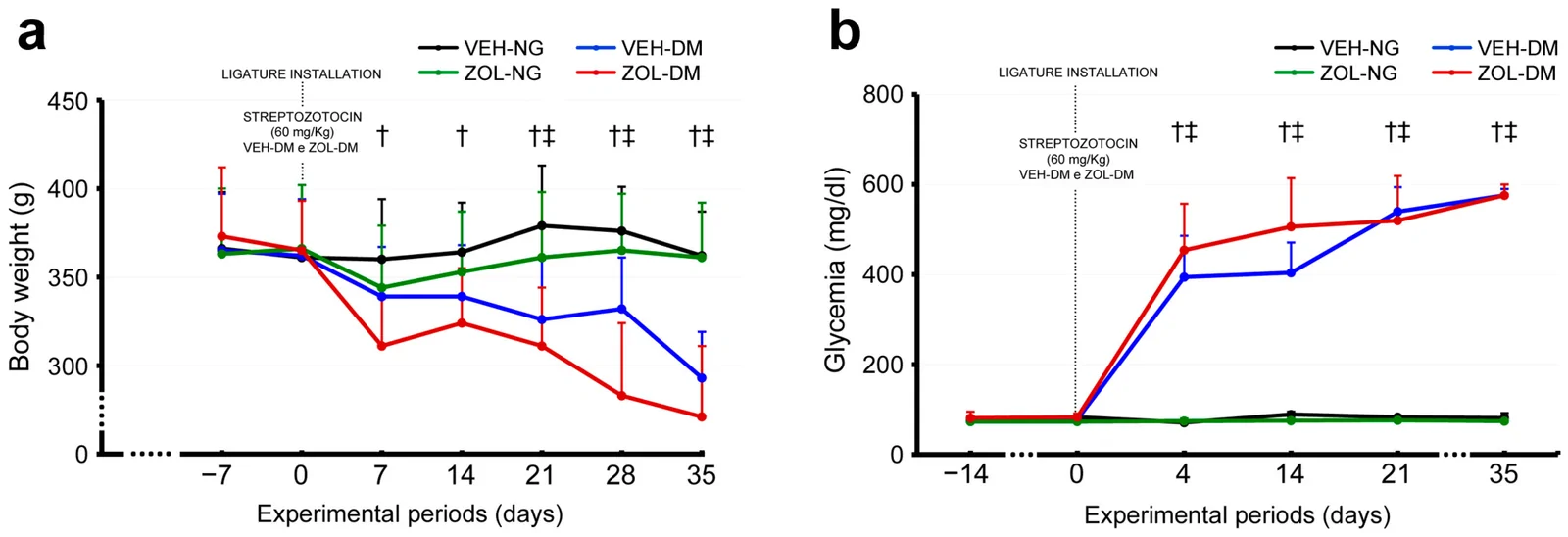
Graphs demonstrating the progression of body weight (**a**) and blood glucose levels (**b**) throughout treatment with an oncological dose of zoledronate in VEH-NG, VEH-DM, ZOL-NG, and ZOL-DM. Note that 14 days after the initiation of vehicle or zoledronate treatment, the VEH-DM and ZOL-DM groups received streptozotocin to induce DM. Symbols: †, statistically significant difference between ZOL-NG and ZOL-DM; ‡, statistically significant difference between VEH-NG and VEH-DM.

There were no significant differences in the final weight between VEH-NG and ZOL-NG. Animals in the VEH-DM and ZOL-DM groups showed a significant decrease in body weight (Figure 2a) and an increase in glycaemic levels after diabetes induction (Figure 2b).

### 3.2. CEJ-ABC, BV/TV, Tb.Th and Tb.N

**Figure 3:**
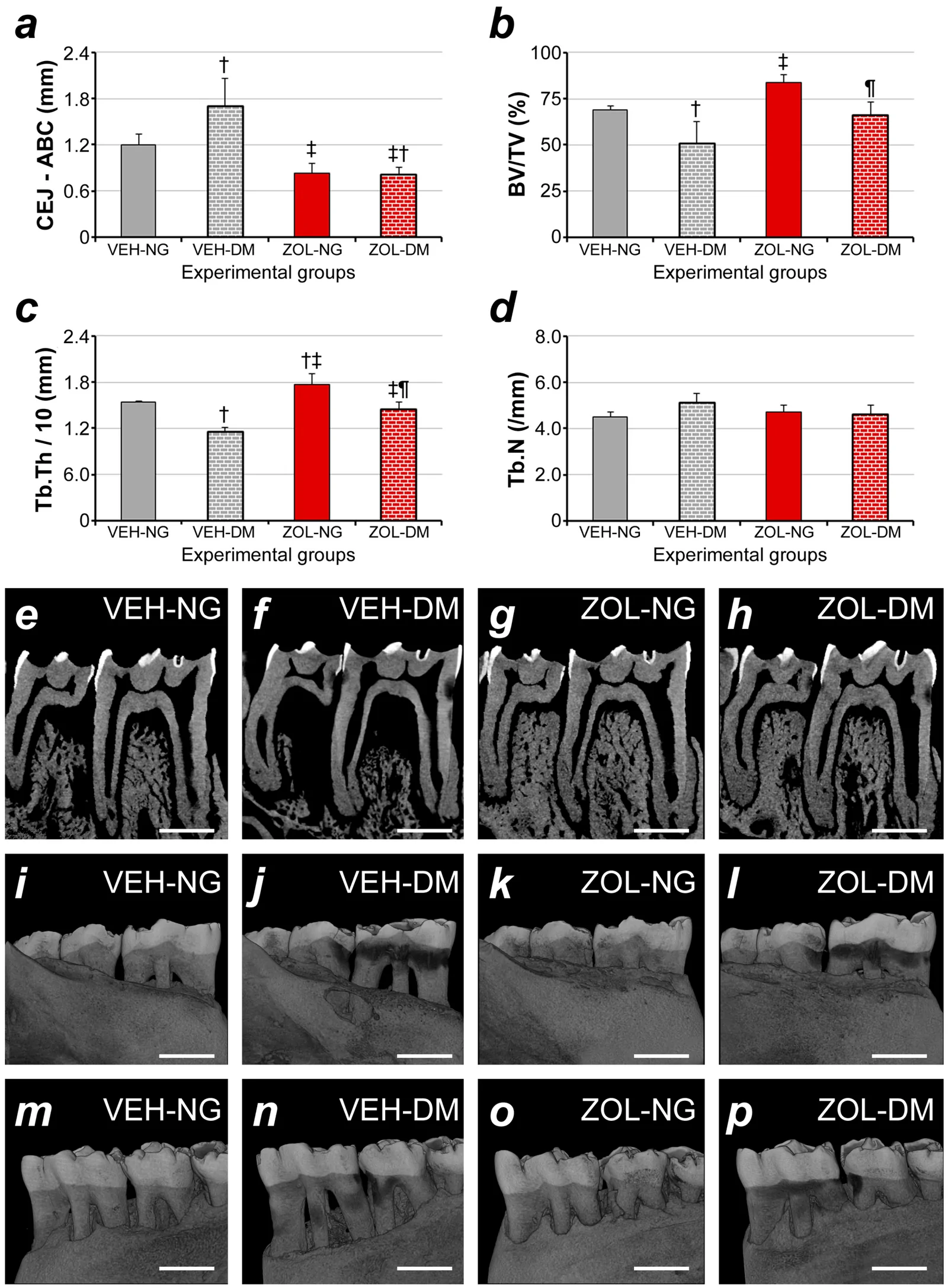
Micro-CT of the mandibular first molar and periodontal attachment apparatus in VEH-NG, VEH-DM, ZOL-NG, and ZOL-DM at 35 days after ligature placement. (**a**–**d**) Graphs showing distance from Cementum enamel junction to Alveolar bone crest (CEJ-ABC) (**a**), alveolar bone volume (BV/TV) (**b**), trabecular thickness (**c**), and trabecular number (**d**) in the furcation region of the mandibular first molar and periodontal attachment apparatus. (**e**–**h**) Two-dimensional micro-CT images of the mandibular first molar and periodontal attachment apparatus in VEH-NG (**e**), VEH-DM (**f**), ZOL-NG (**g**), and ZOL-DM (**h**). (**i**–**p**) Three-dimensional micro-CT reconstructions of the mandibular first molar and periodontal attachment apparatus in VEH-NG (**i**,**m**), VEH-DM (**j**,**n**), ZOL-NG (**k**,**o**), and ZOL-DM (**l**,**p**). Scale bars: 1 mm. Symbols: †, statistically significant difference compared with VEH-NG; ‡, statistically significant difference compared with VEH-DM; ¶, statistically significant difference compared with ZOL-NG.

ZOL-DM presented a significantly lower CEJ-ABC compared to VEH-NG and VEH-DM. There was no statistically significant difference between ZOL-NG and ZOL-DM. VEH-DM showed higher CEJ-ABC compared to VEH-NG (Figure 3a). VEH-DM showed lower BV/TV compared to VEH-NG. ZOL-NG showed higher BV/TV when compared to VEH-DM and ZOL-DM (Figure 3b). ZOL-DM showed greater trabecular thickness compared to VEH-DM. ZOL-NG showed greater trabecular thickness compared to VEH-NG, VEH-DM and ZOL-DM. VEH-DM showed lower trabecular thickness compared to VEH-NG (Figure 3c). There was no statistically significant difference between the VEH-NG, VEH-DM, ZOL-NG and ZOL-DM groups regarding the trabecular number (Figure 3d).

The two- and three-dimensional microtomographic appearance of the insertion periodontium in the different experimental groups is shown in Figure 3.

### 3.3. Histological Characteristics of Periodontal Tissues

**Figure 4:**
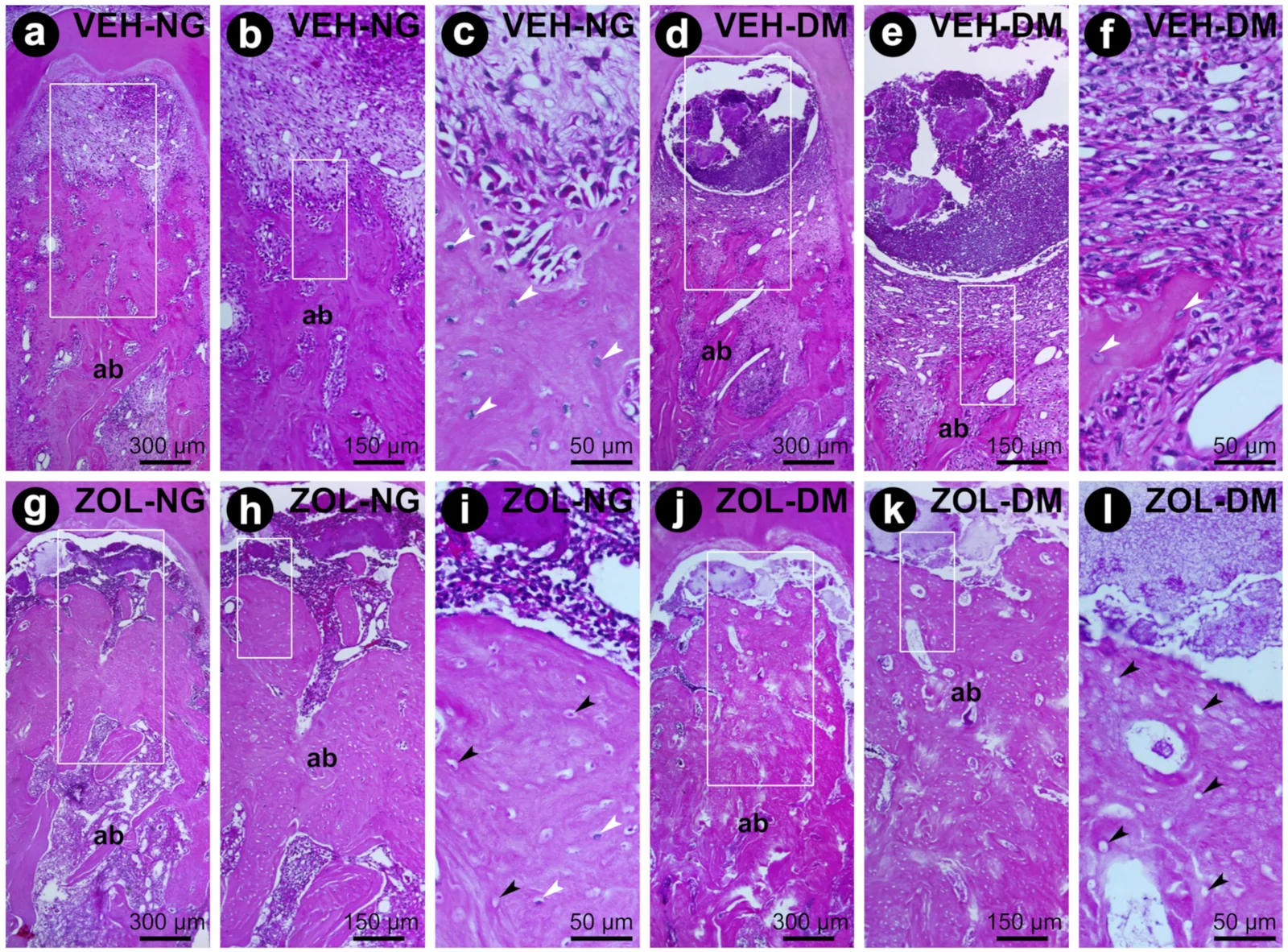
Photomicrographs of furcation region showing the histological appearance of the periodontal attachment apparatus of the mandibular first molar at 35 days after ligature placement in VEH-NG (**a**–**c**), VEH-DM (**d**–**f**), ZOL-NG (**g**–**i**), and ZOL-DM (**j**–**l**). Abbreviations and symbols: ab, alveolar bone; white arrows, osteocytes; black arrows, empty lacunae or lacunae containing necrotic osteocyte remnants. The white rectangles in figures (**a**,**d**,**g**,**h**) represent the regions shown at higher magnification in figures (**b**,**e**,**h**,**k**). The white rectangles in figures (**b**,**e**,**h**,**k**) represent the regions shown at higher magnification in figures (**c**,**f**,**i**,**l**). Original magnification: (**a**,**d**,**g**,**j**) 50×; (**b**,**e**,**h**,**k**) 100×; (**c**,**f**,**i**,**l**) 400×. Scale bars: (**a**,**d**,**g**,**j**) 300 µm; (**b**,**e**,**h**,**k**) 150 µm; (**c**,**f**,**i**,**l**) 50 µm. Staining: H&E.

VEH-DM presented a more severe disease than VEH-NG. ZOL-NG and ZOL-DM exhibited an exaggerated local inflammatory response, minor alveolar bone loss, and a progressive increase in non-vital bone tissue. The periodontal inflammatory response was more pronounced in ZOL-DM than in ZOL-NG. The parameters, scores and distribution of specimens are presented in Table 1 and Figure 4. 

### 3.4. PBTF and PNVBT

**Figure 5:**
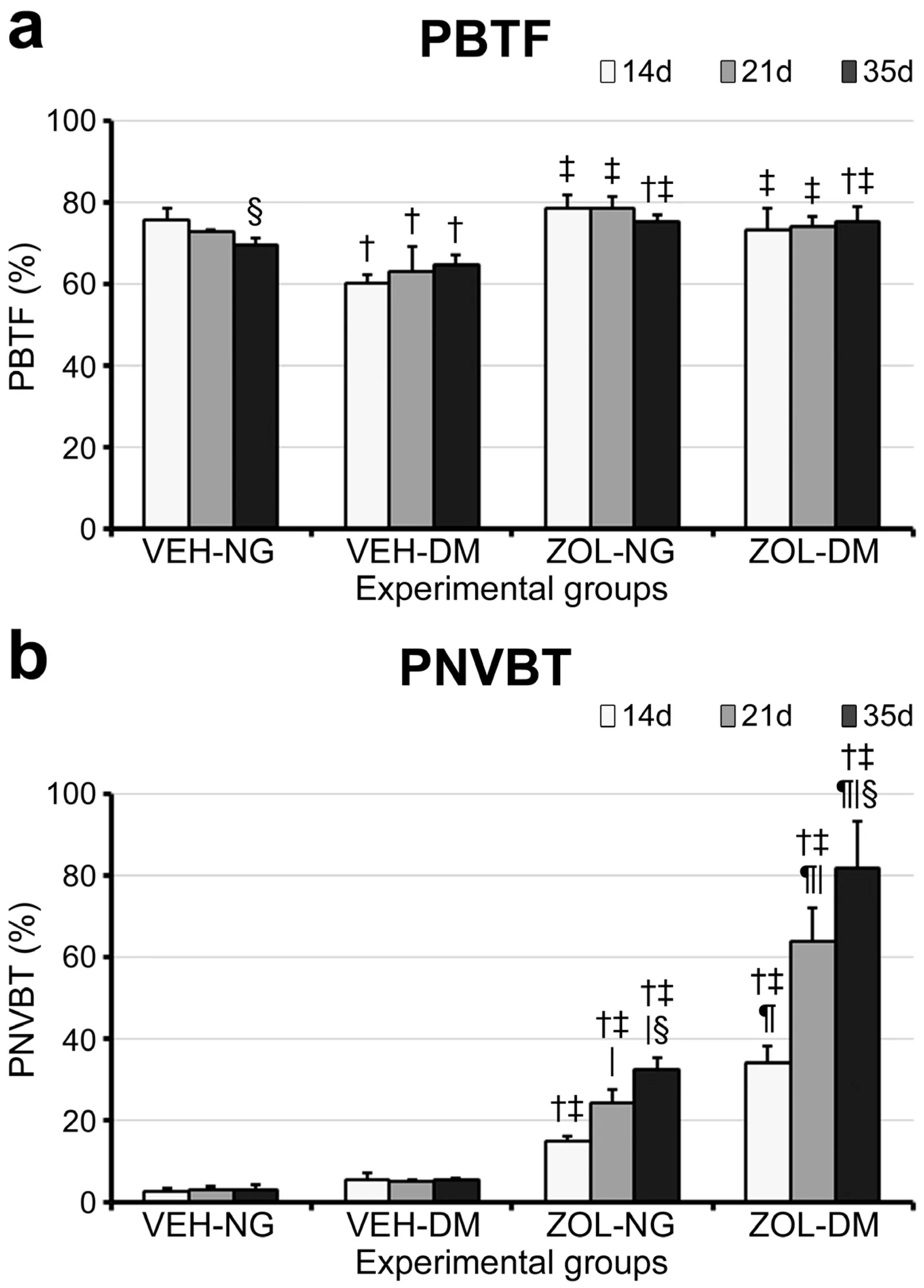
Graphs showing the percentage of bone tissue in the furcation (PBTF) (**a**) and the percentage of non-vital bone tissue (PNVBT) (**b**) in the furcation region of the mandibular first molar at 14, 21, and 35 days after ligature placement in VEH-NG, VEH-DM, ZOL-NG, and ZOL-DM. Statistical test: ANOVA—Tukey. Abbreviations and symbols: †, statistically significant difference compared with VEH-NG at the same time point; ‡, statistically significant difference compared with VEH-DM at the same time point; ¶, statistically significant difference compared with ZOL-NG; ǀ, statistically significant intragroup difference compared with 14 days post-ligature; §, statistically significant intragroup difference compared with 21 days post-ligature.

VEH-DM presented significantly lower PBTF than VEH-NG, ZOL-NG and ZOL-DM in all experimental periods. There was no significant difference between the ZOL-NG and ZOL-DM groups regarding PBTF. ZOL-NG and ZOL-DM showed higher PBTF than VEH-NG 35 days after EP induction. The VEH-NG group showed lower PBTF at 35 days than at 14 days after EP induction (Figure 5a).

In ZOL-NG, PNVBT was significantly higher than in VEH-NG and VEH-DM. ZOL-DM showed higher PNVBT than the other experimental groups. In both ZOL-NG and ZOL-DM, PNVBT was higher at 35 days compared to 14 and 21 days and higher at 21 days compared to 14 days after ligature placement (Figure 5b).

### 3.5. TNFα, IL-1β and TRAP Immunohistochemistry

**Figure 6:**
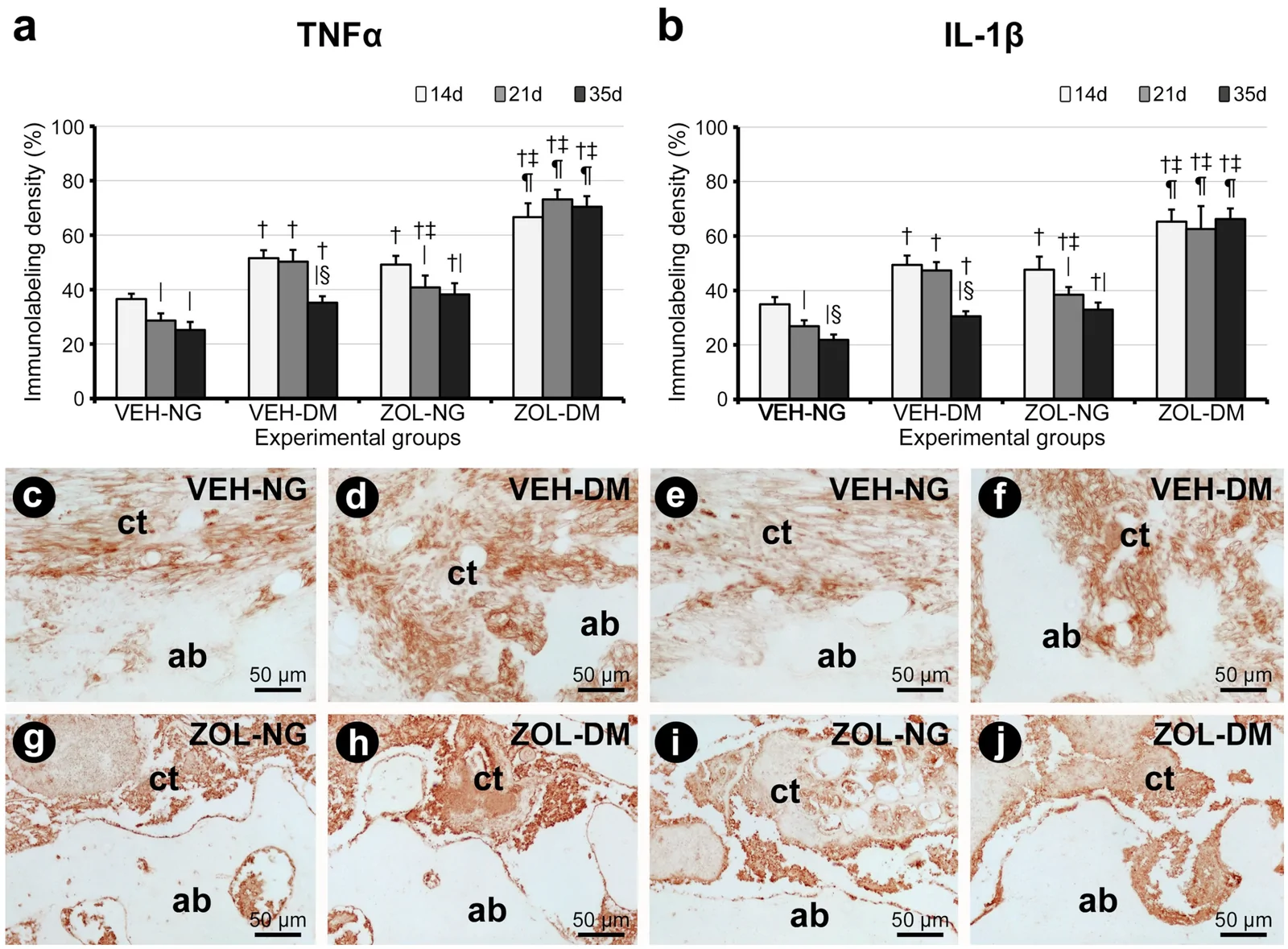
Immunostaining for TNFα and IL-1β in the periodontal attachment apparatus of the mandibular first molar. Graphs showing the optical density of immunostaining for TNFα (**a**) and IL-1β (**b**) in the experimental groups. Representative photomicrographs showing immunostaining for TNFα (**c**,**d**,**g**,**h**) and IL-1β (**e**,**f**,**i**,**j**) in VEH-NG (**c**,**e**), VEH-DM (**d**,**f**), ZOL-NG (**g**,**i**), and ZOL-DM (**h**,**j**) at 14 days after ligature placement. Abbreviations and symbols: ct, connective tissue; ab, alveolar bone; †, statistically significant difference compared with VEH-NG at the same time point; ‡, statistically significant difference compared with VEH-DM at the same time point; ¶, statistically significant difference compared with ZOL-NG; ǀ, statistically significant intragroup difference compared with 14 days post-ligature; §, statistically significant intragroup difference compared with 21 days post-ligature. Original magnification: 400×. Scale bars: 50 µm.

ZOL-DM showed significantly greater immunolabelling for TNFα and IL-1β than the other experimental groups in all periods analysed. The immunolabelling density for both cytokines was higher in VEH-DM and ZOL-NG when compared to VEH-NG in all experimental periods. In VEH-DM at 35 days after ligature placement, immunolabelling for TNFα and IL-1β was lower than at 14 and 21 days. In VEH-NG and ZOL-NG, both at 21 and 35 days, immunolabelling for both cytokines was lower compared to 14 days; in addition, immunolabelling for IL-1β in VEH-DM at 35 days was lower than in the other experimental periods. The immunolabelling pattern for TNFα and IL-1β is depicted in Figure 6.

**Figure 7:**
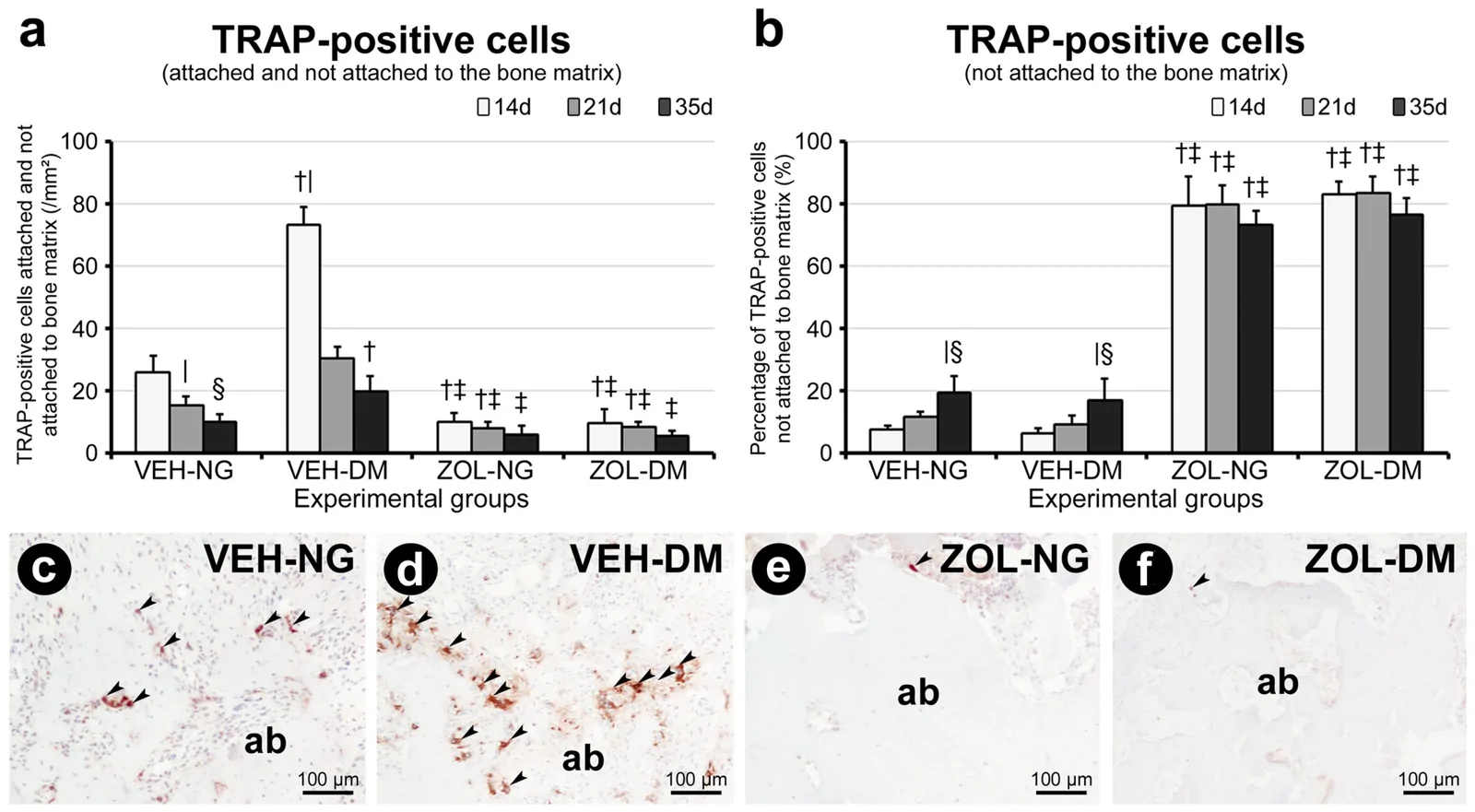
Immunostaining for TRAP in the periodontal attachment apparatus of the mandibular first molar. Graphs showing the number of TRAP-positive cells attached and not attached to the bone matrix (**a**), and the number of TRAP-positive cells not attached to the bone matrix only (**b**), in the experimental groups. Representative photomicrographs showing immunostaining in VEH-NG (**c**), VEH-DM (**d**), ZOL-NG (**e**), and ZOL-DM (**f**) at 14 days after ligature placement. Abbreviations and symbols: ab, alveolar bone; black arrows, TRAP-positive cells; †, statistically significant difference compared with VEH-NG at the same time point; ‡, statistically significant difference compared with VEH-DM at the same time point; ǀ, statistically significant intragroup difference compared with 14 days post-ligature; §, statistically significant intragroup difference compared with 21 days post-ligature. Original magnification: 250×. Scale bars: 100 µm.

VEH-DM presented a higher number of TRAP-positive cells than the other experimental groups in all periods analysed. At 14 and 21 days after ligature placement, VEH-NG presented a higher number of TRAP-positive cells when compared to the groups treated with zoledronate (ZOL-NG and ZOL-DM). In VEH-NG and VEH-DM, the number of TRAP-positive cells was higher at 14 days than at 21 and 35 days. Only in VEH-DM was the number of TRAP-positive cells higher at 21 days compared to 35 days. The vehicle-treated groups (VEH-NG and VEH-DM) showed a significantly greater number of TRAP-positive cells attached to the bone matrix when compared with the zoledronate-treated groups in all experimental periods. The immunolabelling pattern for TRAP is depicted in Figure 7.

## 4. Discussion

Antiresorptive drug therapy, DM, and periodontitis are likely to become increasingly common in the population, considering that this type of treatment and such diseases increase as age advances. The present study was designed to combine several risk factors for MRONJ [1]. Regarding the risk factor related to the medication, zoledronate was used at an oncological dose and 12-month-old female rats were used, considering that MRONJ most frequently affects middle-aged to senescent women [4,5]. Regarding the associated comorbidity, diabetes was induced via a single dose of streptozotocin, considering that this is one of the most common systemic disorders in individuals who developed MRONJ [28]. Regarding the local risk factor, EP was induced via ligature placement to promote plaque accumulation, since EP is the second local risk factor for MRONJ [1].

The present study found, using micro-CT and histometric analyses, that therapy with zoledronate significantly reduces alveolar bone loss during EP. Similarly, other authors also found, using micro-CT analyses, that bone volume fraction was significantly higher in extraction sites of animals treated with zoledronate than controls [29]. Although these results may appear favourable in terms of reducing bone loss during the progression of experimental periodontitis, our histometric results highlighted a deleterious effect of potent inhibition of bone resorption. In ZOL-NG, the PNVBT was significantly higher and even more pronounced in ZOL-DM compared to the VEH-NG and VEH-DM groups. In addition, PNVBT increased throughout the experimental periods in the ZOL-NG and ZOL-DM groups. Corroborating this, other authors have found an increase in non-vital bone tissue caused by treatment with an oncological dose of zoledronate in normoglycaemic rats in EP sites [30,31,32,33]. The use of oncological doses of zoledronate inhibited bone resorption and the consequent bone remodelling process. This may lead to the accumulation of microdamage and non-viable bone tissue in the alveolar bone found in the present study.

The VEH-DM group exhibited a decrease in the magnitude of the inflammatory response, the extent of tissue inflammation, and the levels of key pro-inflammatory cytokines—TNFα and IL-1β—presumably reflecting the transition of the inflammation to a chronic state over time. In contrast, in the ZOL-DM group, inflammation, pro-inflammatory cytokine levels and the PNVBT remained extremely high and unchanged over time. DM and the oncological dose of zoledronate are capable of increasing the levels of cytokines with pro-inflammatory activity [34,35]. It is known that chronic hyperglycaemia favours the formation of advanced glycation end products (AGEs) [36]. The interaction of AGEs with the receptor for advanced glycation end products (RAGE) initiates a cascade of biological events that increase oxidative stress, stimulating the synthesis and accumulation of AGEs and levels of pro-inflammatory cytokines [10,37]. Morita et al. [35] showed high levels of TNFα, IL-1β and IL-6 in an experimental model of MRONJ. In addition, the authors demonstrated that TNFα-, IL-1α/β- or IL-6-deficient mice were significantly resistant to MRONJ. Taken together, our findings suggest a potentially higher risk of MRONJ in diabetics with periodontal disease treated with an oncological dose of zoledronate.

The observed increase in pro-inflammatory cytokines, such as TNFα and IL-1β, likely contributes to the enhanced recruitment of osteoclasts to the site of EP, observed in the VEH-NG group. In the VEH-DM group, the rise in these cytokines led to greater osteoclast recruitment. As these cells were fully active, this resulted in more significant alveolar bone loss in this group. In contrast, groups treated with zoledronate showed a reduced number of osteoclasts at the site of EP, with most of these cells exhibiting characteristics of inactivity in bone resorption. Some studies indicate that osteoclasts and their precursors, when exposed to zoledronate, become important sources of pro-inflammatory cytokines [37,38,39,40]. Additionally, the authors suggest that zoledronate stimulates the polarisation of macrophages to the M1 phenotype [37,38,39,40], which is also involved in producing pro-inflammatory cytokines. Therefore, it is reasonable to link the elevated levels of TNFα and IL-1β in the zoledronate-treated groups to the activity of monocytes, macrophages, and osteoclasts in response to zoledronate treatment.

The alveolar bone at the site of EP is exposed to a microenvironment characterised by high levels of pro-inflammatory cytokines, which may lead to osteocyte death. This cell death primarily occurs through apoptosis and necroptosis. However, the inhibition of bone resorption prevents phagocytic cells from accessing these cellular remains. Consequently, these dead cells can generate damage-associated molecular patterns (DAMPs) that travel through the system of lacunae and canaliculi to the soft tissues adjacent to the bone. This process activates pattern recognition receptors (PRRs) predominantly found in monocytes, macrophages, and osteoclasts, especially in the presence of zoledronate, thereby stimulating these cells to produce even more pro-inflammatory cytokines [31]. In summary, this creates a positive feedback loop that results in additional osteocyte death, escalating the extent of bone necrosis. Furthermore, in the zoledronate-treated diabetic mellitus group, the levels of pro-inflammatory cytokines are significantly higher, primarily due to elevated levels of advanced glycation end-products (AGEs) [36].

While this study did not primarily focus on microbiological factors, it is important to acknowledge that the site affected by EP has a complex microbiota [41,42]. This microbiota likely contributes to the worsening of the inflammatory condition and may lead to increased bone resorption or, in cases where this process is inhibited by zoledronate, it could promote osteocyte death. Furthermore, when osteocytes die and bone resorption is inhibited, the lacunae and canaliculi present in the alveolar bone create a favourable environment for the growth and spread of microorganisms. To date, it has not been definitively established whether infection plays a primary or secondary role in the development of MRONJ [43,44]. However, its influence requires further investigation.

Our findings demonstrated that, in diabetic rats treated with an oncological dose of zoledronate, the severity of experimental periodontitis was significantly increased, thereby predisposing the site to MRONJ. Although this study did not reveal clinical manifestations of MRONJ in the animals, it is important to highlight that we used a precise method, histomorphometric analysis, to evaluate its hallmark feature: bone necrosis. This method provides more accurate measurement of the lesions, including at an earlier stage. Experimental animal models can develop lesions that resemble MRONJ and exhibit similar clinical, imaging, and histopathological characteristics, although they may not be identical to those observed in humans. Therefore, while MRONJ diagnosis in humans primarily relies on clinical features, diagnosis in experimental animal models, particularly in rats, can be based primarily on the histopathological characteristics of the lesions.

Treatment with antiresorptive drugs requires continuous, coordinated monitoring by a multidisciplinary team. Maintaining optimal dental and periodontal health is essential throughout therapy. In this situation, periodontitis should be considered a key warning sign, as it can act as a local risk factor for MRONJ, as demonstrated in the present study. Likewise, DM warrants careful attention—even when periodontal tissues appear healthy—because it is a systemic comorbidity known to substantially increase MRONJ risk, especially when associated with a local risk factor, even though our study did not evaluate DM in isolation from periodontitis. The findings of the present study clearly show that the coexistence of periodontitis and poor glycemic control markedly amplifies the likelihood of developing MRONJ. This condition remains a significant clinical challenge because its etiopathogenesis is not yet fully understood, and multiple local and systemic factors influence the onset, severity, and progression of the disease. The consequences can be severe, and treatment outcomes are often unpredictable or unsatisfactory. While the results of this preclinical animal study cannot be directly extrapolated to humans, they reinforce the importance of strict glycemic control and vigilant periodontal monitoring during and after antiresorptive therapy.

## 5. Conclusions

During treatment with an oncological dose of zoledronate, periodontitis and glycaemic dyscontrol substantially increase the risk of MRONJ.

## Figures and Tables

**Table 1:** Parameters, scores, and distribution of the specimens based on the histopathological analyses of the furcation region of mandibular left molars in the VEH-NG, VEH-DM, ZOL-NG and ZOL-DM groups.

Histopathological Analyses												
Parameters and Scores	Samples											
	Experimental Groups											
	VEH-NG			VEH-DM			ZOL-NG			ZOL-DM		
	14 d	21 d	35 d	14 d	21 d	35 d	14 d	21 d	35 d	14 d	21 d	35 d
**Intensity of Local Inflammatory Response**												
(1) absence of inflammation	-	-	-	-	-	-	-	-	-	-	-	-
(2) small quantity of inflammatory cells (less than 1/3 of cells were inflammatory cells)	-	-	-	-	-	-	-	-	-	-	-	-
(3) moderate quantity of inflammatory cells (1/3 to 2/3 were inflammatory cells)	5	8	8	-	-	-	-	-	-	-	-	-
(4) large quantity of inflammatory cells (more than 2/3 were inflammatory cells)	3	-	-	6	6	8	8	8	8	-	-	-
(5) extremely large quantity of inflammatory cells	-	-	-	2	2	-	-	-	-	8	8	8
**Extension of Inflammatory Infiltrate**												
(1) absence of inflammation	-	-	-	-	-	-	-	-	-	-	-	-
(2) partial extension of connective tissue in the furcation region	-	-	-	-	-	-	-	-	-	-	-	-
(3) entire extension of connective tissue in the furcation region	6	8	8	-	8	8	-	-	-	-	-	-
(4) entire extension of connective tissue and partial extension of bone tissue in the furcation region	2	-	-	8	-	-	8	8	8	-	-	-
(5) entire extension of connective tissue and bone tissue in the furcation region	-	-	-	-	-	-	-	-	-	8	8	8
**Pattern of Structuration of the Connective Tissue in the Furcation Region**												
(1) moderate number of fibroblasts and large amount of collagen fibres (dense connective tissue)	-	-	-	-	-	-	-	-	-	-	-	-
(2) moderate amount of both fibroblasts and collagen fibres	-	-	-	-	-	-	-	-	-	-	-	-
(3) small amount of both fibroblasts and collagen fibres	6	6	8	2	2	2	-	-	-	-	-	-
(4) partial tissue disorganisation with necrosis areas	2	2	-	6	6	6	8	8	8	-	-	-
(5) severe tissue disorganisation with necrosis areas	-	-	-	-	-	-	-	-	-	8	8	8
**Pattern of Structuration of the Alveolar Bone in the Furcation Region**												
(1) Bone trabeculae with regular contours, lined by osteoblasts and rare osteoclasts	-	-	-	-	-	-	-	-	-	-	-	-
(2) Bone trabeculae with irregular contours, lined by both osteoblasts and active osteoclasts	-	-	-	-	-	-	-	-	-	-	-	-
(3) Bone trabeculae with irregular contours, containing numerous active osteoclasts	8	8	8	8	8	8	-	-	-	-	-	-
(4) Moderate amount of non-vital (necrotic) bone tissue	-	-	-	-	-	-	5	6	8	-	-	-
(5) High amount of non-vital (necrotic) bone tissue	-	-	-	-	-	-	3	2	-	8	8	8

## Data Availability

The raw data supporting the conclusions of this article will be made available by the authors on request.
